# scNAT: a deep learning method for integrating paired single-cell RNA and T cell receptor sequencing profiles

**DOI:** 10.1186/s13059-023-03129-y

**Published:** 2023-12-18

**Authors:** Biqing Zhu, Yuge Wang, Li-Ting Ku, David van Dijk, Le Zhang, David A. Hafler, Hongyu Zhao

**Affiliations:** 1 Program of Computational Biology and Bioinformatics, Yale University, New Haven, CT 06511 USA; 2 Department of Biostatistics, School of Public Health, Yale University, New Haven, CT 06511 USA; 3 Department of Internal Medicine, Yale School of Medicine, New Haven, CT 06511 USA; 4 Department of Computer Science, Yale University, New Haven, CT 06511 USA; 5 Department of Neuroscience, School of Medicine, Yale University, New Haven, CT 06511 USA; 6 Department of Neurology, School of Medicine, Yale University, New Haven, CT 06511 USA; 7 Department of Immunobiology, School of Medicine, Yale University, New Haven, CT 06511 USA; 8 Aligning Science Across Parkinson’s (ASAP) Collaborative Research Network, Chevy Chase, USA MD 20815

**Keywords:** scRNA-seq, scTCR-seq, Data integration, Deep learning, Variational autoencoder, Clone expansion

## Abstract

Many deep learning-based methods have been proposed to handle complex single-cell data. Deep learning approaches may also prove useful to jointly analyze single-cell RNA sequencing (scRNA-seq) and single-cell T cell receptor sequencing (scTCR-seq) data for novel discoveries. We developed scNAT, a deep learning method that integrates paired scRNA-seq and scTCR-seq data to represent data in a unified latent space for downstream analysis. We demonstrate that scNAT is capable of removing batch effects, and identifying cell clusters and a T cell migration trajectory from blood to cerebrospinal fluid in multiple sclerosis.

**Supplementary Information:**

The online version contains supplementary material available at 10.1186/s13059-023-03129-y.

## Background

The rapid development of next-generation sequencing (NGS) technologies has enabled researchers to detect complex signals in biological samples, especially at the single-cell resolution through single-cell RNA sequencing (scRNA-seq) and multi-omics [1, 2]. With the generation of massive scRNA-seq data, it is challenging to use traditional linear statistical methods to extract complex patterns in these data. In contrast, deep learning-based methods have been widely employed thanks to their capability of modeling non-linear features [3], ability to conduct transfer learning across different domains [4], and scalability in terms of data size [5], to handle single cell data for different tasks, such as gene imputation [6, 7], batch correction [8–10], cell clustering [11, 12], TCR sequence classification [13], expression program identification [14], and multi-omics data integration [15]. For instance, SAUCIE [9] is an autoencoder designed to perform multiple tasks simultaneously, including data denoising, imputation, visualization, and clustering via various layers of regularizations, including information dimension regularization and maximal mean discrepancy correction. scGNN [6] is a graph neural network (GNN) model which conducts gene imputation and cell clustering iteratively that is regularized by a left-truncated mixture Gaussian model to account for cell-type-specific signals. Moreover, by learning a joint probabilistic distribution of the paired RNA and protein data, totalVI [16] employs a GNN model to jointly analyze the cellular indexing of transcriptomes and epitopes by sequencing (CITE-seq) data comprising data integration, joint dimension reduction, and missing protein imputation.

In parallel to scRNA-seq technologies, high-throughput single cell T cell receptor sequencing (scTCR-seq) has been developed to characterize the complex composition of immune repertoires [17, 18]. The TCR of more than 95% of all T cells consists of two chains, TCR$\alpha$ and TCR$\beta$ [19]; TCR$\alpha$ is encoded by variable (V) and joining (J) gene segments, and TCR$\beta$ by variable (V), diversity (D), and joining (J) segments. The diversity of the TCR repertoires is characterized by the V/(D)/J gene rearrangement and the variability in the complementarity determining region 3 (CDR3) encoded by the V/(D)/J junction [20]. Specifically, the CDR3 is hypervariable for both the TCR$\alpha$ and TCR$\beta$ chains, and responsible for peptide recognition presented by the cell surface major histocompatibility complex (MHC) protein. scTCR-seq technology has enabled profiling of the diversity of the TCR repertoire, quantify the clonal expansion of T cells in the adaptive immune response [21], and characterize the associations between TCR and T cell subtypes [22].

Analyzing paired scRNA-seq and scTCR-seq data in various biological domains has led to insight into disease pathogenesis. For example, one study found that in multiple sclerosis (MS), genes related to T cell cytotoxic function and activation were upregulated in the clonally expanded T cells from cerebrospinal fluid (CSF) [23]. Similarly, clonal expansion of CD$8^+$ T cells was found in the CSF of patients with Alzheimer’s disease [24]. A growing number of methods have been proposed for multi-omics data integration. For example, MOFA+ [25] integrates single-cell multi-modal data by variational inference and reconstructs a low-dimensional data embedding. scAI [26] performs an integrative analysis on transcriptomic and epigenomic data through iterative learning, employing a deep generative probabilistic model. MultiVI [27] integrates different single-cell level data modalities using a deep generative model for probabilistic analysis. However, these methods were specifically designed for numerical data, posing difficulties in their applications to TCR data that contains CDR3 and V/(D)/J gene features, which are considered as text and categorical data, respectively. On the other hand, recent methods have been developed to integrate TCR information or infer the correlation between scRNA-seq and scTCR-seq data. For example, DeepTCR [13] leverages neural networks to integrate V/(D)/J gene and CDR3 data to facilitate downstream analyses such as dimension reduction and clustering. CoNGA [28] is a graph theoretic approach for calculating the correlation between scRNA-seq data and the corresponding TCR sequence data for new cell type discovery. Tessa [29] is a Bayesian model integrating TCRs with T cell gene expression data to better understand the T cell phenotypes. However, DeepTCR only focuses on TCR data and completely neglects RNA modality which contains transcription-level information. CoNGA takes into account RNA data but it still visualizes RNA and TCR data via separate UMAP representations. Furthermore, it collapses all the cells within the same clonotype, resulting in the loss of singe-cell-level resolution. Tessa does not take into consideration the CDR3$\alpha$ and V/(D)/J gene information, and similar to CoNGA, tessa assumes that all the cells having the same clonotype share the same transcriptomic profile, which is unrealistic. Therefore, there is a lack of systematic approaches which jointly consider the two data types as a whole for data integration, although previous studies have revealed that T cells within the same clonotype share similar transcriptome profiles [22, 30]. Pappalardo et al. have also observed this phenomenon in the T cells from the blood of patients with MS [23], with cells within the same clonotype closer to each other in the UMAP plots, indicating similar gene expression patterns. Here, we introduce scNAT, a deep learning method for integrating paired **sc**R**NA**-seq and sc**T**CR-seq profiles, to learn patterns in these two data types through a unified latent space to facilitate downstream analyses. With scNAT, we were able to identify a cluster of T cells that are in transition state as well as a T cell migration trajectory from blood to CSF in an MS dataset.

## Results

### scNAT model specification

**Fig. 1:**
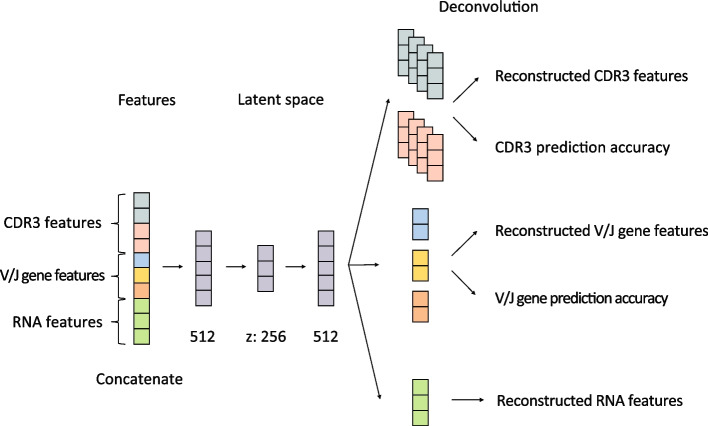
A variational autoencoder (VAE) model built from the concatenated features. The CDR3, V/J gene, and RNA features were first concatenated and then treated as input to the VAE in order to learn the underlying structure

Our method is based on a variational autoencoder (VAE) to integrate both scRNA-seq and paired scTCR-seq data as well as to facilitate scalable unsupervised learning. The input consists of three parts: CDR3 features from scTCR-seq data, V/J gene features from scTCR-seq data, and RNA features from scRNA-seq data. To enable VAE integration, we first transform the $\alpha$- and $\beta$-chain CDR3 variable from a discrete sequence into a continuous numerical space through a one-layer neural network followed by a three-layer convolutional neural network (CNN) (Fig. S1C). Similarly, V/J genes in both chains are provided as categorical variables and first encoded into a one-hot encoding representation and then transformed into a continuous vector by leveraging a trainable embedding layer (Fig. S1B). Additionally, the top 2000 highly variable genes are selected from the RNA data, and the dimension is then reduced by a fully connected layer to be more comparable with the scTCR-seq data (Fig. S1A). Afterwards, the three preprocessed data vectors are concatenated as input to the VAE model to learn the underlying data structure informed by both the RNA and TCR data. The model has three layers, the middle of which is the latent space parametrized by a multivariate normal distribution. Then, these data are reconstructed through deconvolution or fully connected layers (Fig. 1). After the model is trained, the integrated features representing both the scRNA-seq and the scTCR-seq information can be extracted from the latent space to facilitate downstream analyses, such as trajectory analysis or clustering. The details of each step are provided in the “Methods” section.

### Application to multiple sclerosis data

**Fig. 2:**
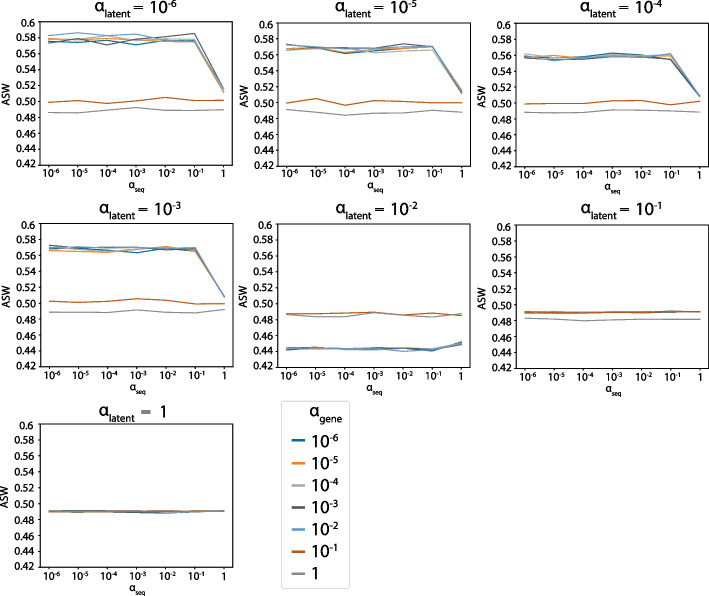
ASW grid search results on the MS dataset. The ASW under different $\alpha _{latent}$, $\alpha _{gene}$, and $\alpha _{seq}$

**Fig. 3:**
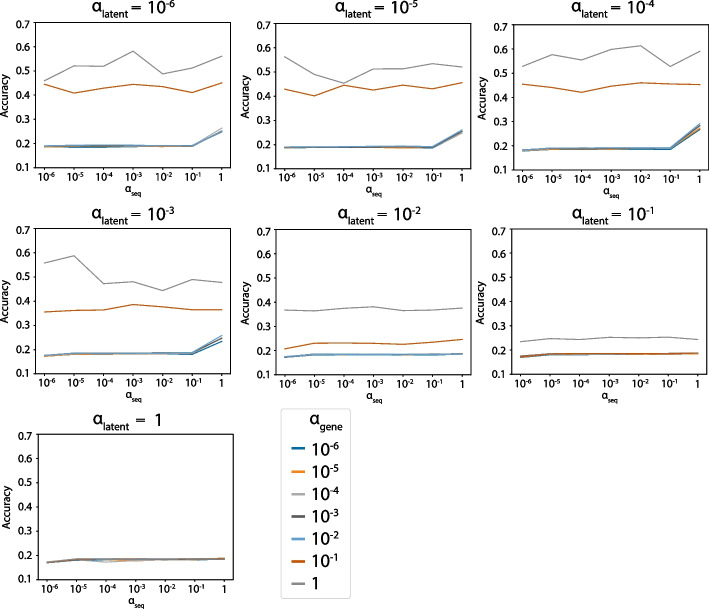
Accuracy grid search results on the MS dataset. V/J gene and CDR3 prediction accuracy under different $\alpha _{latent}$, $\alpha _{gene}$, and $\alpha _{seq}$

**Fig. 4:**
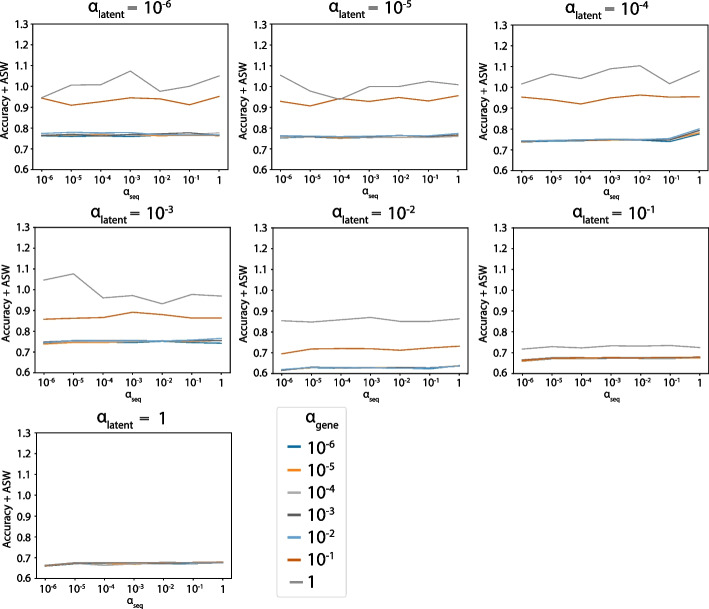
ASW and accuracy grid search results on the MS dataset. The sum of ASW and accuracy under different $\alpha _{latent}$, $\alpha _{gene}$, and $\alpha _{seq}$

Multiple sclerosis (MS) is a chronic inflammatory demyelinating disease that affects the central nervous system. MS is genetically mediated involving several immune cells and is primarily driven by T cells [31, 32]. To reveal the details of the immune processes in MS, we applied scNAT to an MS dataset in Pappalardo et al. [23], containing six healthy controls and five patients with MS. For each individual, both blood and cerebrospinal fluid (CSF) were sequenced. In total, there were 48,898 cells, including CD$4^+$ naive T cell, CD$4^+$ memory T cell, CD$8^+$ naive T cell, CD$8^+$ memory T cell, and regulatory T cell (T$_{reg}$) in blood as well as CD$4^+$ memory T cell, CD$8^+$ memory T cell, and T$_{reg}$ in CSF (Table S1, Fig. S2). To determine the weight parameters for the loss function, we first conducted an extensive grid search on different parameter combinations (Methods, Figs. 2, 3, 4, and S3). Then, these parameters were applied to scNAT as hyperparameters for training and the latent features were extracted to facilitate downstream analysis.

#### scNAT latent features represent information from both the scRNA-seq as well as the scTCR-seq data

**Fig. 5:**
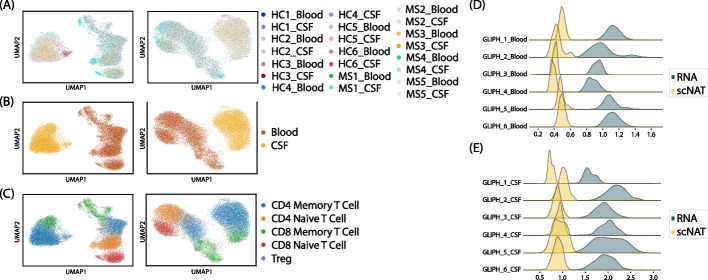
Latent space and GLIPH2 comparison on the MS dataset. **A**–**C** ResPAN (RNA) UMAP (left) and scNAT UMAP (right) colored by batch (**A**), source (**B**), and cell type (**C**). **D**, **E** Pair-wise normalized Euclidean distance comparison of the GLIPH2 clusters between RNA and scNAT in blood (**D**) and CSF (**E**)

We first compared the UMAP (Methods) calculated by scRNA-seq data only (Fig. 5A–C right), scTCR-seq data only (Fig. S4A), scNAT integrated data using normalized and batched corrected RNA data (Fig. 5A–C left), and scNAT integrated data using RNA raw count data (Fig. S4B). The results showed that the scNAT integrated data with normalized and batch corrected input can preserve tissue and cell type information as the RNA data, but TCR data themselves do not provide direct information in terms of cell types. On the other hand, scNAT with RNA raw count input also struggled with learning the underlying biological structure due to early stopping, which shows the importance of data normalization (Methods). We further investigated the performances of DeepTCR [13], tessa [29], and CoNGA [28] (Methods, Fig. S5). There is no obvious pattern in the tessa TCR network to reflect biological information (Fig. S5A), and tessa was not able to retain much cell type-specific or tissue-specific information through the weighted TCR embedding (Fig. S5B-C). Similar to scNAT with scTCR-seq only data, DeepTCR results also preserve very little cell type or tissue information (Fig. S5D–E). This is expected since the structure of the TCR part of scNAT is similar to DeepTCR. On the other hand, since CoNGA reduced data into one cell per clonotype, the information from UMAP of the scRNA-seq data is very limited compared with the UMAP from the full scRNA-seq data (Figs. S5F-G, 5B-C left). And similar to tessa and DeepTCR, the CoNGA TCR UMAP structure does not reflect cell source information (Fig. S5H–I). The run time and memory information is summarized in Table S2. Taken together, we have further demonstrated that by integrating scRNA-seq and scTCR-seq data into a united latent space, scNAT can achieve better performance in terms of cell type and tissue information preservation.

Next, to quantify to which extent scNAT can preserve the information from TCR modality, GLIPH2 [33] was applied to identify T cell clusters by CDR3 similarity (Methods). We selected the six largest GLIPH2 clusters across blood and CSF which had high CD8 percentage (Fig. S6A–C). The results demonstrate that the normalized pair-wise distances within GLIPH2 clusters in the scNAT integrated space are much smaller than those in the RNA space for both tissue types (Fig. 5D, E, Methods), suggesting the clonotype-related information is also incorporated into scNAT integrated data.

#### scNAT can further remove remaining batch effects

We first demonstrate that scNAT can better remove batch effects than ResPAN. As stated in the previous section, both methods can distinguish blood and CSF (Fig. 5B), and the corresponding cell types in each source are clearly separated in the UMAP plots (Fig. 5C). However, in ResPAN, the batch effects from the CSF in healthy control 3 (HC3) and blood in MS 4 (MS4) were still largely preserved, but in the scNAT processed data, all samples were better mixed (Fig. 5A). To further quantify the batch removal performance, we considered two metrics, 1 − bASW [34] and 1 − kBET [35], where higher scores indicate better batch correction performance (Methods). The results showed that scNAT achieved 16% higher 1 − kBET score than ResPAN (0.387 versus 0.333), and a slightly higher 1 − bASW score (0.566 versus 0.561) (Table 1). We also compared scNAT with popular batch correction approaches designed for scRNA-seq data, including Seurat v4 [36], MNN [37], and BBKNN [38], and found out that scNAT consistently performed the best among these methods (Table 1). These  results demonstrate that apart from data integration, scNAT can further remove remaining batch effects.

**Table 1:** Batch correction scores comparing scNAT and other methods

Method	1 − bASW	1 − kBET
scNAT	0.566	0.387
ResPAN	0.561	0.333
Seurat v4	0.550	0.267
MNN	0.561	0.263
BBKNN	0.557	0.224

#### Characterization of the T cell trafficking trajectory from blood to CSF

**Fig. 6:**
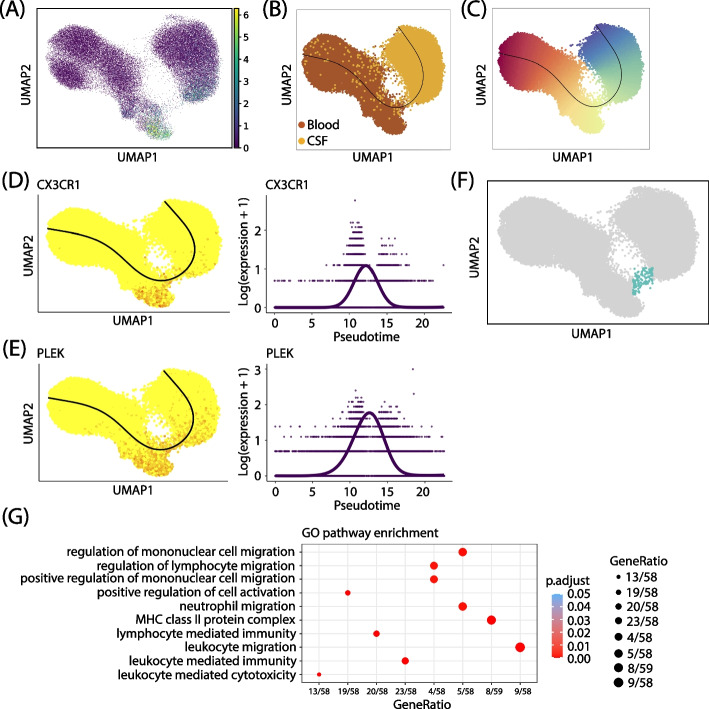
A T cell trafficking trajectory from blood to CSF identified by scNAT. **A** scNAT UMAP colored by expansion score. **B**, **C** Slingshot inferred trajectory colored by tissue (**B**) and pseudotime (**C**). **D**, **E** Genes that are associated with the trajectory identified by TradeSeq in UMAP (left) and the corresponding smoothers (right). **F** A cluster of T cells that are in transitioning state. **G** Upregulated GO pathways of the transitioning cluster compared with other cells

Since previous studies have found that T cells can enter the CSF through the choroid plexus for immune surveillance [23, 39], we interrogated if the same observation can be made for the MS dataset to understand the molecular mechanism. To this end, we defined expansion score as in Pappalardo et al. [23], which is the log$_2$ of the number of cells in a clonal group and corresponds to the clone size, and found that CD8$^+$ memory T cells in both blood and CSF had the highest score, suggesting this cell type was undergoing clonal expansion (Fig. 6A). Further, to examine whether the CD8$^+$ memory T cells in the two sources came from the same clonal group, we selected a few large clones and found that the predominance of the cells overlapped with the cells with higher expansion scores in both blood and CSF (Fig. S7A), consistent with the known observation that CD8$^+$ memory T cells may enter the CSF from blood. To further confirm this observation, we applied Slingshot [40] to infer the cell lineage and pseudotime, and the results showed that there is indeed a trajectory of T cells from blood to CSF with a small cluster of transitioning CD8$^+$ memory T cells in the middle (Fig. 6B–C, F, Methods). The application of Monocle3 [41, 42] resulted in similar patterns (Fig. S8A-B). A trajectory was identified from blood CD8 memory T cells to CSF, with the increase of pseudotime. To further validate this finding, we applied the same procedure using only the RNA data (Fig. S8C-G), and found that the RNA-only data could partially recover the pattern, with the cells having high expansion score being the CD8 memory T cells towards the high end of the UMAP for both blood and CSF (Fig. S8C-D), and with the cells still migrating from blood to CSF (Fig. S8E-F). However, the RNA data failed to bridge the two tissues, rather the cells that should be in transitioning state were pushed towards the boundaries of blood and CSF (Fig. S8G). Given the trajectory, we then studied which genes are associated with the trajectory to better understand the migration mechanism. We applied tradeSeq [43] to infer genes that were differentially expressed along the trajectory (Methods). Overall, we identified 50 DE genes (Figs. 6D-E, S7B-C), and the full gene list can be found in Additional file 2: Table S3. Among these genes, CX3CR1 has been reported to prevent remyelination in a cuprizone model of demyelination [44], underscoring its neuroprotective effects [45] (Fig. 6D). PLEK was identified as an MS risk gene from both RNA [46] and GWAS studies [47] (Fig. 6E). Next, we focused on the small cluster that was in the transitioning state (Fig. 6F). To further corroborate our hypothesis that these cells are migrating and clonally expanded T cells, we examined the distribution of the CDR3$\beta$ chain sequence in this cluster as well as the distribution of the chain in random cells and found that the CDR3 in the transitioning cluster was more homogeneous than the one in random cells of the same size (Fig. S7D). Finally, the upregulated GO pathways of this cluster with respect to all the other cells (Additional files 3 and 4: Table S4-5) can be broadly divided into two groups, including cell migration and leukocyte cytotoxicity (Fig. 6G), which suggests that the cells were migrating from blood to CSF and were clonally expanded due to activation. Overall, this indicates that T cells in MS can transmigrate from blood to CSF and some of the transitioning cells are under clonal expansion, showing signatures of T cell cytotoxicity.

## Discussion

Next-generation sequencing generates vast amounts of molecular data, especially in the field of genomics and transcriptomics, which requires advanced computational and analytical methods to extract meaningful insights and improve our understanding of biological processes. Deep learning has been applied extensively in this field to analyze and interpret these complex data. In this paper, we present scNAT, a deep learning-based model to integrate scRNA-seq and the paired scTCR-seq data for the purpose of novel cell population identification and biological process inference. To the best of our knowledge, our method represents the first approach capable of integrating the three complex data types, including numerical, text, and categorical data in order to perform this task. While previous methods including MOFA+, scAI, and LIGER are capable of integrating multi-omics data, they fail to handle data types other than numerical data. On the other hand, tessa ignores CDR3$\alpha$ as well as V/(D)/J gene information, and assumes all the cells within the same clone have the same RNA profile, which resulted in unstructured data (Fig. S5B–C). In contrast, DeepTCR combines the CDR3 sequences and V/(D)/J gene usage to characterize the TCR data, but it does not take into account RNA information which is a valuable data source for cell type identification and functional characterization of different cell types (Fig. S5D–E). Similarly, although the CoNGA algorithm computes correlations between RNA and TCR data, it does not render a joint representation of the two data modalities, hindering downstream visualization and analysis (Fig. S5F–I).

Through both gird search and real data analysis, we demonstrate that scNAT can balance the RNA and TCR modalities in a data-driven manner, achieved through loss function hyperparameter tuning. While we only explore the integration of the TCR and RNA data, this framework can be extended to include other data modalities such as single-cell BCR-seq [48] and single cell immune profiling with other modalities such as cell surface marker. By utilizing deep learning, it is possible to create a rich feature space that can incorporate multiple data types, providing greater flexibility in data modeling. We demonstrated the performance of scNAT through its application to an MS dataset. We showed that with leveraging information from the TCR data, the resulting UMAP can not only preserve the cell type information but can also incorporate signals from the clonotype data. The application suggests that apart from data integration, scNAT can further remove remaining batch effect from the input data which have been preprocessed by some batch correction method. Moreover, through integrating different data modalities, scNAT can also benefit other downstream analyses, such as trajectory inference.

We note a few limitations in our pipeline. The first is the huge variability in both TCR $\alpha$ and $\beta$ chains. Due to the randomness in the V/(D)/J combination and the addition or deletion at the CDR3 junction site, it is difficult to perform systematic simulation to evaluate model performance under different scenarios. Furthermore, the black-box nature of the neural network hampers our ability to interpret the results. For example, although there are hyperparameters in the loss function controlling the weight of each modality, understanding which RNA or TCR contributes to the final result of each cell is still challenging.

## Conclusions

We demonstrate that the use of deep learning to integrate scRNA-seq and scTCR-seq data allows for a more comprehensive analysis that leverages the strengths of both data types. By combining gene expression information from scRNA-seq with TCR repertoire diversity and clonality information from scTCR-seq, we can better understand the complex immune response and identify novel cell populations that may have been previously overlooked. The resulting integrated data can also be used for trajectory inference, which allows for the study of cellular differentiation and development over time. This approach is the first to handle numerical, text, and categorical data types all together in order to integrate RNA and TCR data, representing a significant advance in the field of single-cell analysis and has the potential to lead to new insights into the immune system and its role in health and disease.

## Methods

### scTCR-seq data curation

scTCR-seq data in the filtered_contig_annotations.csv files were collected from the two data sources as described in the manuscript. Only T cells with $\alpha$ and $\beta$ chains were kept and we further chose cells with both RNA and paired TCR data.

### scRNA-seq data preprocessing

scRNA-seq data were processed in accordance with the scanpy [49] (v1.7.2, RRID:SCR_018139) workflow for preprocessing. Next, ResPAN [10] (v0.1.0) was applied to remove batch effects. Cell type annotation was achieved via domain knowledge and canonical marker genes.

### scNAT architecture

#### Data transformation

scNAT takes normalized, log-transformed scRNA-seq gene expression with the top 2000 highly variable genes as input. Further, to be more comparable with the dimension of the paired scTCR-seq data, the scRNA-seq input was transformed through a fully connected layer of length 1024 (Fig. S1A). Each V/J gene is considered as a categorical input to the model and was first converted to a one-hot embedding and then transformed into a continuous numeric space by a trainable embedding layer of dimension 48. Finally, all the gene features for both TCR$\alpha$ and TCR$\beta$ were concatenated to represent the joint feature of the V/J gene usage for each T cell (Fig. S1B). For the CDR3 sequence, similar to the V/J gene, the amino acid sequence was first transformed to a one-hot embedding and then to a continuous numeric vector. Subsequently, three CNN layers with the feature maps 32, 64, and 128 were added to extract sequences and allow all the sequences to share the same sets of NN parameters. Detailed structure can be found in Sidhom et al. [13]. Finally, the outputs for both TCR$\alpha$ and TCR$\beta$ were flattened and concatenated as the CDR3 representation (Fig. S1C). For the TCR data integration (Fig. S4A), only the V/J gene and CDR3 information were input to the model.

#### VAE structure

The core of the model is a VAE which is able to extract features from the scRNA-seq data, V/J gene usage, and the CDR3 sequences. The three data types were first concatenated to get an overall continuous representation (*X*) of the multi-omics data, and then a VAE model with layer sizes of 512, 256, and 512 was applied in order to learn the low dimensional distribution of the combined data by a multi-dimensional standard Gaussian distribution parametrization. Finally, the sampled data from the latent space were reconstructed through fully-connected and deconvolutional layers (Fig. 1).

### Training VAE

To train the neural network, we implemented the Adam Optimizer with learning rate 0.001 in order to minimize both the variational loss and the reconstruction loss. The variational loss is the Kullback-Leibler (KL) divergence between the encoder’s distribution and a unit Gaussian:

1 $$\begin{aligned} V_{loss} = D_{KL}(N(\mu (X), \sigma (X) || N(0, 1))). \end{aligned}$$

The reconstruction loss has three parts. The first component is the RNA reconstructed loss which is defined as the average of the mean squared error between the original RNA input ($X_g$) and the reconstructed RNA data ($\hat{X_g}$) across all *G* genes:

2 $$\begin{aligned} R_{RNA} = \frac{1}{G}\sum \limits _{g}\left( X_{g} - \hat{X}_{g}\right) ^2. \end{aligned}$$

The second part is the V/J gene cross-entropy loss between the one-hot encoded input V/J gene usage ($T_{orig}$) and the reconstructed gene ($T_{recon}$) across all the gene positions (*V* and *J*) and the two chains ($\alpha$ and $\beta$):

3 $$\begin{aligned}&R_{gene} = -\left( \sum \limits _{m} T_{orig,m} log(T_{recon, m}) + \sum \limits _{n} T_{orig,n} log(T_{recon, n})\right) ; m \in \{V_{\alpha }, J_{\alpha }\},\nonumber \\&n \in \{V_{\beta }, J_{\beta }\}. \end{aligned}$$

The third piece is the reconstruction loss of the CDR3 sequence represented by the cross-entropy loss between the one-hot encoded CDR3 input ($S_{orig}$) and the reconstructed sequence ($S_{recon}$) across the two chains:

4 $$\begin{aligned} R_{CDR3} = -(S_{orig, \alpha } log(S_{recon, \alpha }) + S_{orig, \beta } log(S_{recon, \beta })). \end{aligned}$$

The total loss is:

5 $$\begin{aligned} L = \alpha _{latent} V_{loss} + \alpha _{gene} R_{gene} + \alpha _{seq} R_{CDR3} + R_{RNA}, \end{aligned}$$

where $\alpha _{latent}$, $\alpha _{gene}$, and $\alpha _{seq}$ are the weights for the variational loss, V/J gene loss, and CDR3 loss, which can be tuned to balance different data modalities as described in the next section. The training with raw RNA counts as input was stopped early after two epochs because there were extreme values in the RNA count matrix, causing infinite values of the weights.

### Grid search for parameter tuning

In order to find the optimal combination of the three weights, we conducted grid search to maximize the sum of the normalized mean Silhouette Coefficient of all samples as well as the V/J gene and CDR3 prediction accuracy. We can define a cell type silhouette score for each cell *i* with $s_{cell type}^{(i)}$, where:

6 $$\begin{aligned} s_{cell type}^{(i)} = \frac{b(i) - a(i)}{max\{a(i), b(i)\}}, \end{aligned}$$

here *a*(*i*) is the mean distance from cell *i* to all other cells that belong to the same cell type, *b*(*i*) is the lowest average distance from cell *i* to all cells in the same cell type among all other cell types.

The normalized Silhouette coefficient quantifies how well the integrated data preserve the cell type information and is defined as:

7 $$\begin{aligned} ASW = \frac{1}{2} \left( \frac{\sum \nolimits _{i = 1}^N s_{cell type}^{(i)}}{N} + 1\right) , \end{aligned}$$

where *N* is the total number of cells, and the normalization ensures the ASW is between 0 and 1.

The predicted V/J gene was set to be the one that corresponds to the index with the largest value of the reconstructed data. Similarly, each amino acid in the predicted CDR3 sequence was determined by the index with the largest value of the reconstructed CDR3 sequence in each position. Finally, the accuracy was computed by comparing the predicted data with the original data. The V/J gene and CDR3 prediction accuracy reflects the weight of the TCR modality.

The parameter grid was set to be $[10^{-6}, 10^{-5}, 10^{-4}, 10^{-3}, 10^{-2}, 10^{-1}, 1]$ for $\alpha _{latent}$, $\alpha _{gene}$, and $\alpha _{seq}$, and during each run, we randomly subsampled 4000 cells to make it computationally efficient. In addition to maximizing the sum of the ASW and accuracy, we (1) put a constraint on ASW such that the resulting value is larger than 0.5 to ensure the performance is better than random, and (2) only selected the parameters whose corresponding loss does not exceed 115% of the minimal loss so that the model fits the data well. The final parameters are $\alpha _{latent} = 10^{-6}$, $\alpha _{gene} = 10^{-3}$, $\alpha _{seq} = 10^{-2}$ for the MS dataset.

### Other methods details

Here, we present the full analysis of the comparisons to other methods referenced in the main text. DeepTCR (v2.1.27) was trained under the default parameters within the unsupervised learning setting. For tessa, due to its high computational cost, only 25% of the cells were used to perform the analysis. We first constructed TCR networks that took into account the association with the scRNA-seq data. After that, we calculated UMAP following the steps in the next section. CoNGA analysis was conducted by first computing TCRdist [50] kernel PCs. After that, the scRNA-seq data were reduced to a single cell per TCR clonotype. Finally, the UMAP was computed for RNA and TCR separately.

### UMAP calculation

The UMAP from scRNA-seq data only was calculated following the standard scanpy [49] pipeline. For the scNAT integrated data, we first ran PCA based on the latent space representation, and then we further embedded the dimension reduced data into the UMAP space. By setting the model parameter *include_RNA* to *False*, the model only included the TCR data when doing the integration for the scTCR-seq data only version, and the UMAP was calculated the same way as the scNAT integrated data.

### GLIPH2 analysis

In order to cluster TCRs based on shared similarity of the CDR3 sequences, GLIPH2 [33] was applied to the MS scTCR-seq data. GLIPH2 clusters TCRs based on global similarity, computed by CDR3 sequences differing by up to one amino acid, and motif-based local similarity, determined by shared enriched CDR3 motifs. The GLIPH2 human CD48 dataset was used for reference. The filtering criteria for GLIPH2 clusters were set to be groups with significant V-gene bias ($P < 0.005$ by GLIPH2) and significant final score ($P < 10^{-5}$ by GLIPH2). The normalized pair-wise distances within each cluster were calculated by dividing the original distance by the mean pair-wise distances across all cells in the RNA PCA or scNAT latent space.

### Batch correction metrics

To evaluate the batch correction performance for scNAT and ResPAN, we considered two metrics: bASW (ASW [34] on batch labels) and kBET [35].

#### bASW

For each tissue, the bASW was computed on batch labels and scaled to ensure it is between 0 and 1 using the equation below:

8 $$\begin{aligned} bASW_{tissue} = \frac{ASW_{tissue} + 1}{2}, \end{aligned}$$

then we weighted the bASW$_{tissue}$ score from blood and CSF based on their cell number $n_{tissue}$:

9 $$\begin{aligned} bASW = bASW_{blood} \frac{n_{blood}}{n_{blood} + n_{CSF}} + bASW_{CSF} \frac{n_{CSF}}{n_{blood} + n_{CSF}}. \end{aligned}$$

Finally, to make sure higher scores correspond to better batch removal performance, the score is scaled by subtracting it from 1:

10 $$\begin{aligned} 1 - bASW. \end{aligned}$$

#### kBET

To identify if there is any bias in a replicated experiment, kBET determines whether the label distribution of a k nearest neighborhood of a cell is similar to the global label distribution by a $\chi ^2$-based test. The test is repeated for random neighborhoods with a fixed size, and the results are summarized by averaging the binary test results to render an overall rejection rate. Since a lower rejection rate indicates well-mixed replicates, similar to the bASW, we use $1 -$kBET to ensure higher score correspond to better mixing performance.

### Trajectory inference

In order to infer the cell trafficking trajectory and underlying pseudotime in the MS dataset, we applied the R (v4.3.2, RRID:SCR 001905) package Slingshot [40] (v3.18, RRID:SCR 017012) and specified the starting cluster and the endpoint. In addition, given the Slingshot trajectory, the method tradeSeq [43] was further implemented to fit a regression model which is able to identify genes that are differentially expressed along this lineage through the R function associationTest. The threshold for DE genes was set to be the Bonferroni corrected *p* value < 0.05 and the mean log fold change > 3. For Monocle3 [41, 42] (v3.14, RRID:SCR 018685), a principal graph from the reduced dimension space was first learned from reversed graph embedding, and then the cells were assigned a pseudotime based on their projection on the principal graph.

### Supplementary Information

**Additional file 1.**

**Additional file 2: Table S3.** Differentially expressed genes identified by tradeSeq in MS dataset.

**Additional file 3: Table S4.** Differentially expressed genes in transitioning cells in MS dataset.

**Additional file 4: Table S5.** Gene Ontology canonical pathways in transitioning cells in MS dataset.

**Additional file 5.** Review history.

## Data Availability

Data for all CSF scRNA-seq and single-cell TCR sequencing from the MS study is available through NCBI’s dbGaP at accession number phs002222.v1.p1 [51]. Python (v3.6.15, RRID:SCR 008394) source code and scNAT is uploaded on https://github.com/biqing-zhu/scNAT [52] - DOI: 10.5281/zenodo.8341925 [53]. The repository is released under the MIT license.
