# Supplementary material for: scNAT: a deep learning method for integrating paired single-cell RNA and T cell receptor sequencing profiles
Source: Genome Biol. 2023 Dec 18;24:292. doi: 10.1186/s13059-023-03129-y (PMC10726524; doi:10.1186/s13059-023-03129-y)
Supplement: Supplementary file 1 — Additional file 1. [file 13059_2023_3129_MOESM1_ESM.pdf]

METHOD

scNAT: a deep learning method for integrating paired single cell RNA and T cell receptor sequencing profiles

Biqing Zhu[1], Yuge Wang[2], Li-Ting Ku[2], David van Dijk[3,4], Le Zhang[5,7], David A. Hafler[6,7] and Hongyu Zhao[1,2*]

*Correspondence:
hongyu.zhao@yale.edu
[2]Department of Biostatistics, School of Public Health, Yale University, 06511, New Haven, CT, USA
Full list of author information is available at the end of the article

Author details
[1]Program of Computational Biology and Bioinformatics, Yale University, 06511, New Haven, CT, USA.
[2]Department of Biostatistics, School of Public Health, Yale University, 06511, New Haven, CT, USA. [3]Department of Internal Medicine, Yale School of Medicine, 06511, New Haven, CT, USA. [4]Department of Computer Science, Yale University, 06511, New Haven, CT, USA. [5]Department of Neuroscience, School of Medicine, Yale University, 06511, New Haven, CT, USA. [6]Department of Immunobiology, School of Medicine, Yale University, 06511, New Haven, CT, USA. [7]Department of Neurology, School of Medicine, Yale University, 06511, New Haven, CT, USA.

References
Supplementary Figures

Supplementary Tables

Supplementary Table 1: MS dataset summary. Frequency and percentage for each cell type in each condition from blood and CSF.

Source	Condition	Cell type	Frequency	Percentage
Blood	Control	$CD4^+$ Memory T Cell	4,147	31.89%
		$CD4^+$ Naive T Cell	4,018	30.89%
		$CD8^+$ Memory T Cell	2,399	18.45%
		$CD8^+$ Naive T Cell	1,870	14.38%
		Treg	572	4.40%
	MS	$CD4^+$ Memory T Cell	4,129	29.07%
		$CD4^+$ Naive T Cell	4,091	28.80%
		$CD8^+$ Memory T Cell	2,620	18.44%
		$CD8^+$ Naive T Cell	2,819	19.84%
		Treg	547	3.85%
CSF	Control	$CD4^+$ Memory T Cell	5,839	84.98%
		$CD8^+$ Memory T Cell	864	12.57%
		Treg	168	2.45%
	MS	$CD4^+$ Memory T Cell	11,047	74.57%
		$CD8^+$ Memory T Cell	3,113	21.01%
		Treg	655	4.42%

Supplementary Table 2: Run time and memory comparison between scNAT and other methods.

Method	Run time	Memory
scNAT	8min50s	1.14GB
Tessa	>3day	NA
Tessa (25% data)	21hr8min20s	8.47GB
DeepTCR	1min16s	1.36GB
CoNGA	1min52s	394.59MB

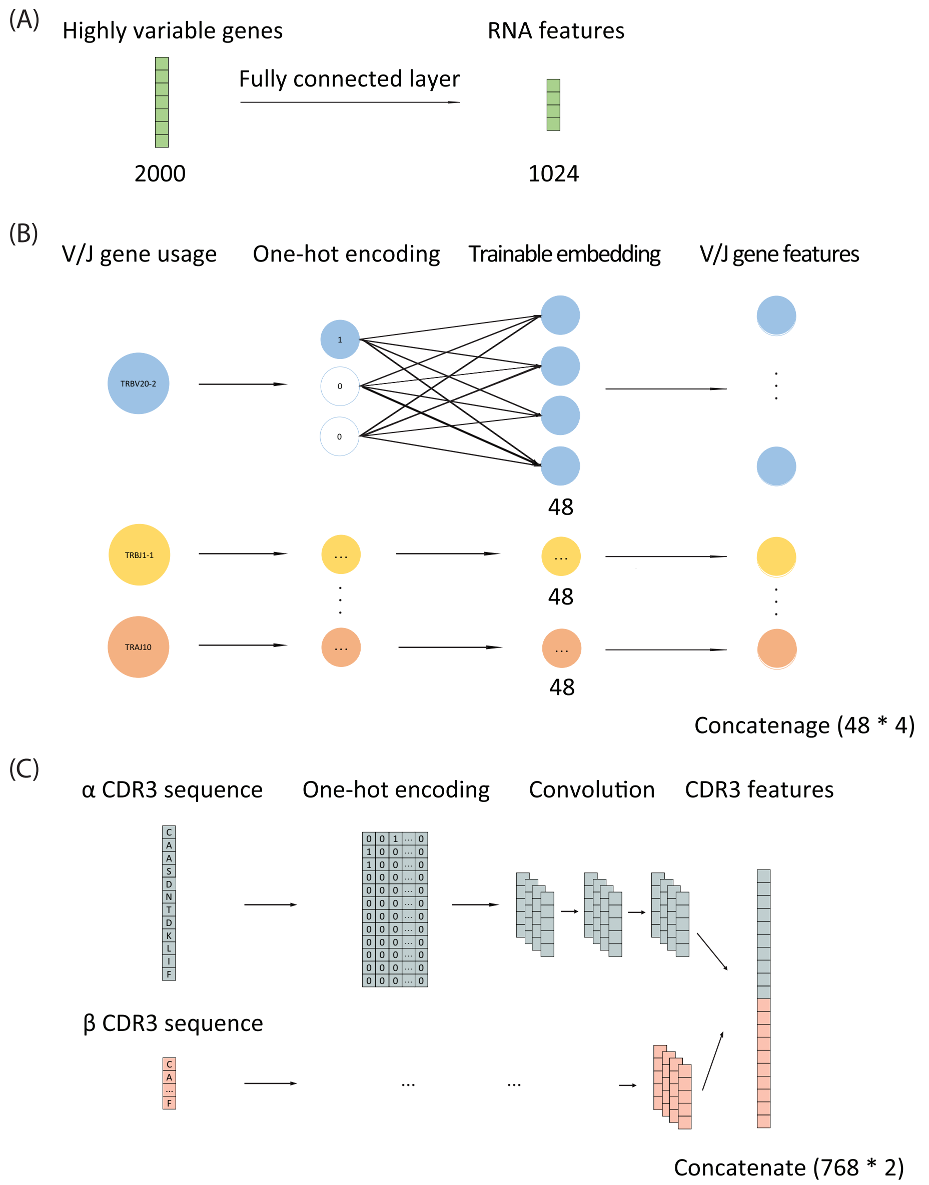

Supplementary Figure 1: **Embedding of scRNA-seq data, V/J genes, and CDR3 sequences.** (A) Transformation of scRNA-seq data through a fully connected layer. (B) Conversion of V/J gene usage into a continuous representation through one-hot encoding, embedding, and concatenation. (C) Application of one-hot encoding, convolution, and concatenation to CDR3 sequences.

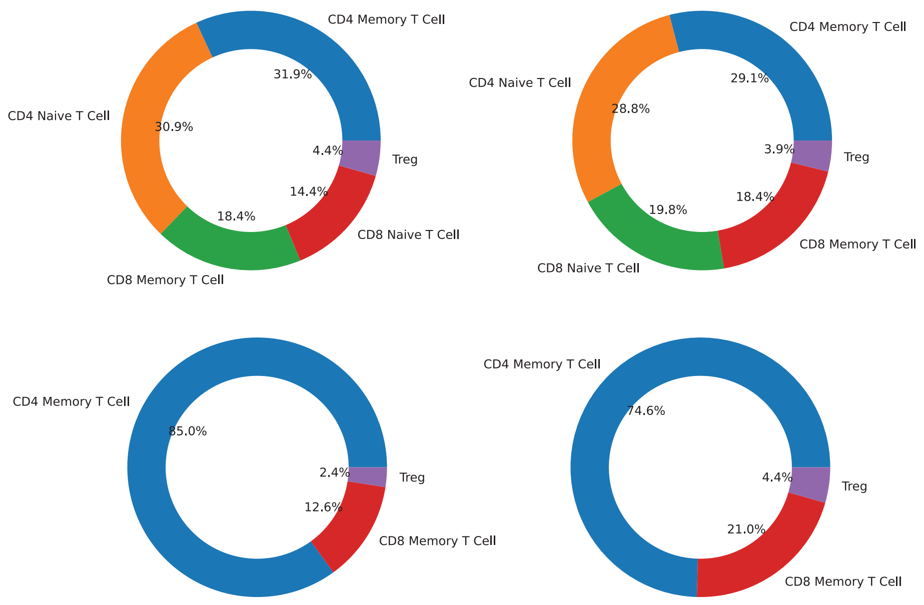

Supplementary Figure 2: **Cell type proportions in blood and CSF.** Cell type proportion in control blood (upper left), MS blood (upper right), control CSF (lower left), and MS CSF (lower right).

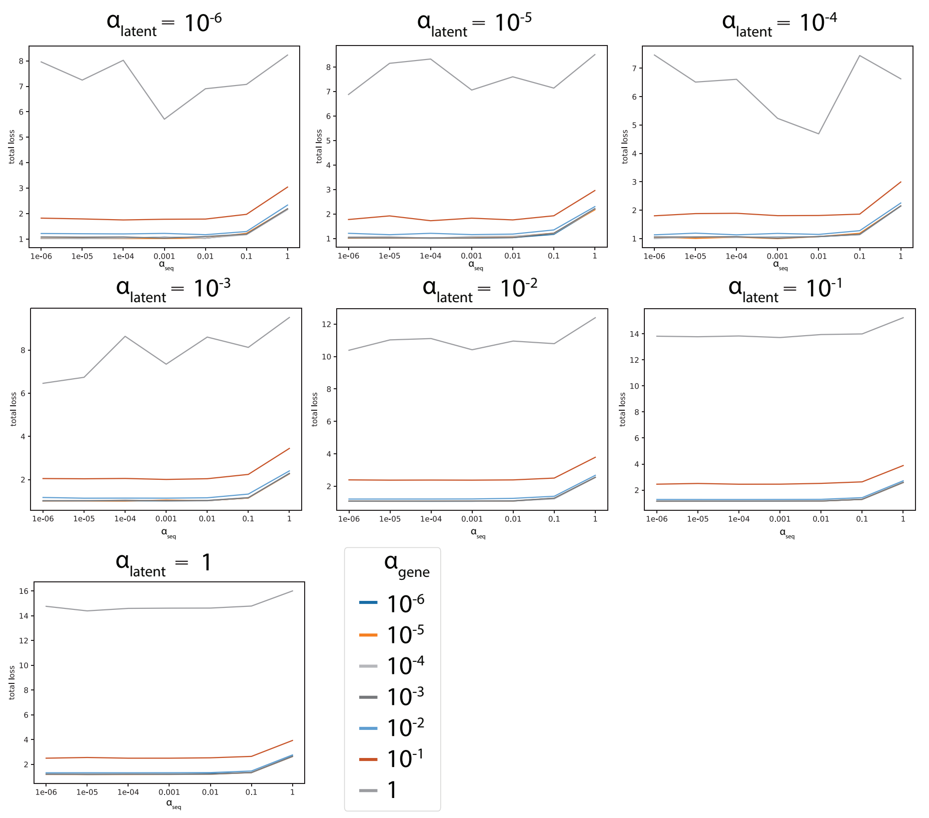

Supplementary Figure 3: **Total loss for grid search results on the MS dataset.** The total loss under different α_{latent}, α_{gene}, and α_{seq}.

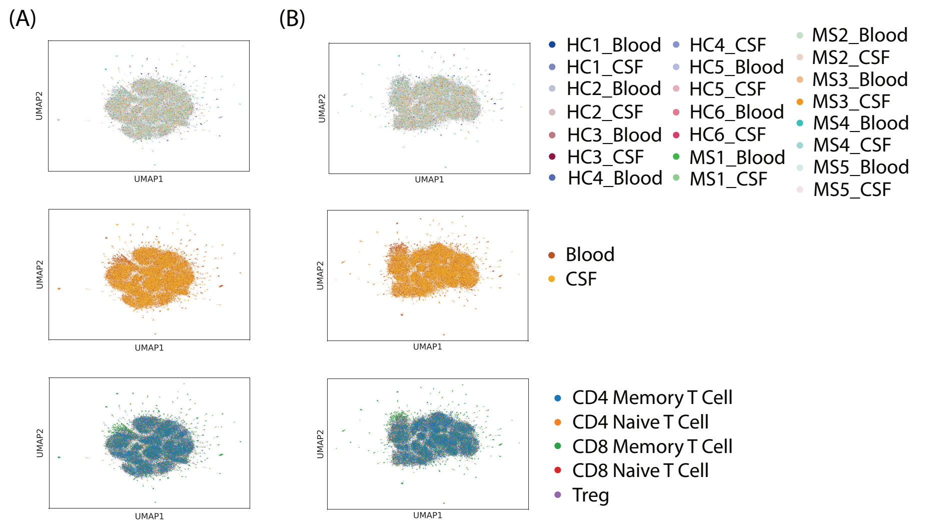

Supplementary Figure 4: **UMAP plots on the MS data.** (A) TCR UMAP colored by batch, source, and cell type. (B) scNAT UMAP calculated using raw count data and colored by batch, source, and cell type.

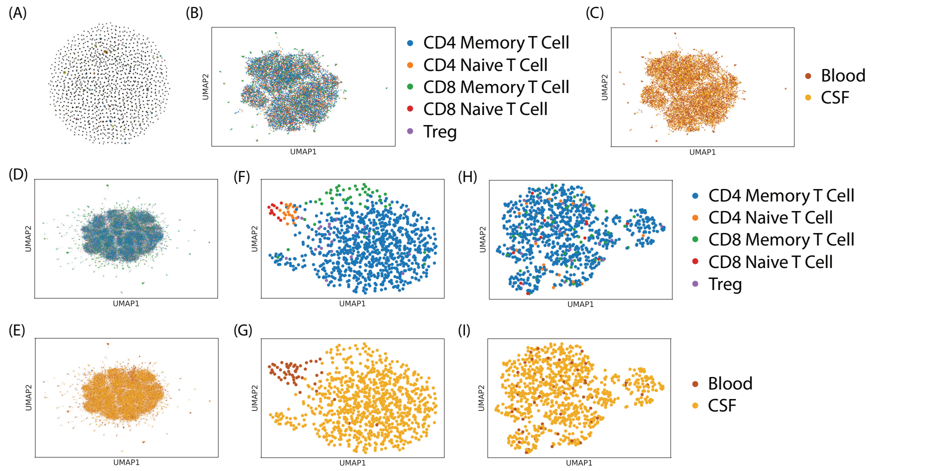

Supplementary Figure 5: **Results from tessa, DeepTCR, and CoNGA.** (A) TCR networks identified by tessa. (B-C) UMAP of the tessa weighted TCR embedding colored by cell type (B) and source (C). (D-E) UMAP of the DeepTCR latent features colored by cell type (D) and source (E). (F-G) UMAP of the CoNGA reduced scRNA-seq data colored by cell type (F) and source (G). (H-I) UMAP of the CoNGA reduced scTCR-seq data colored by cell type (H) and source (I).

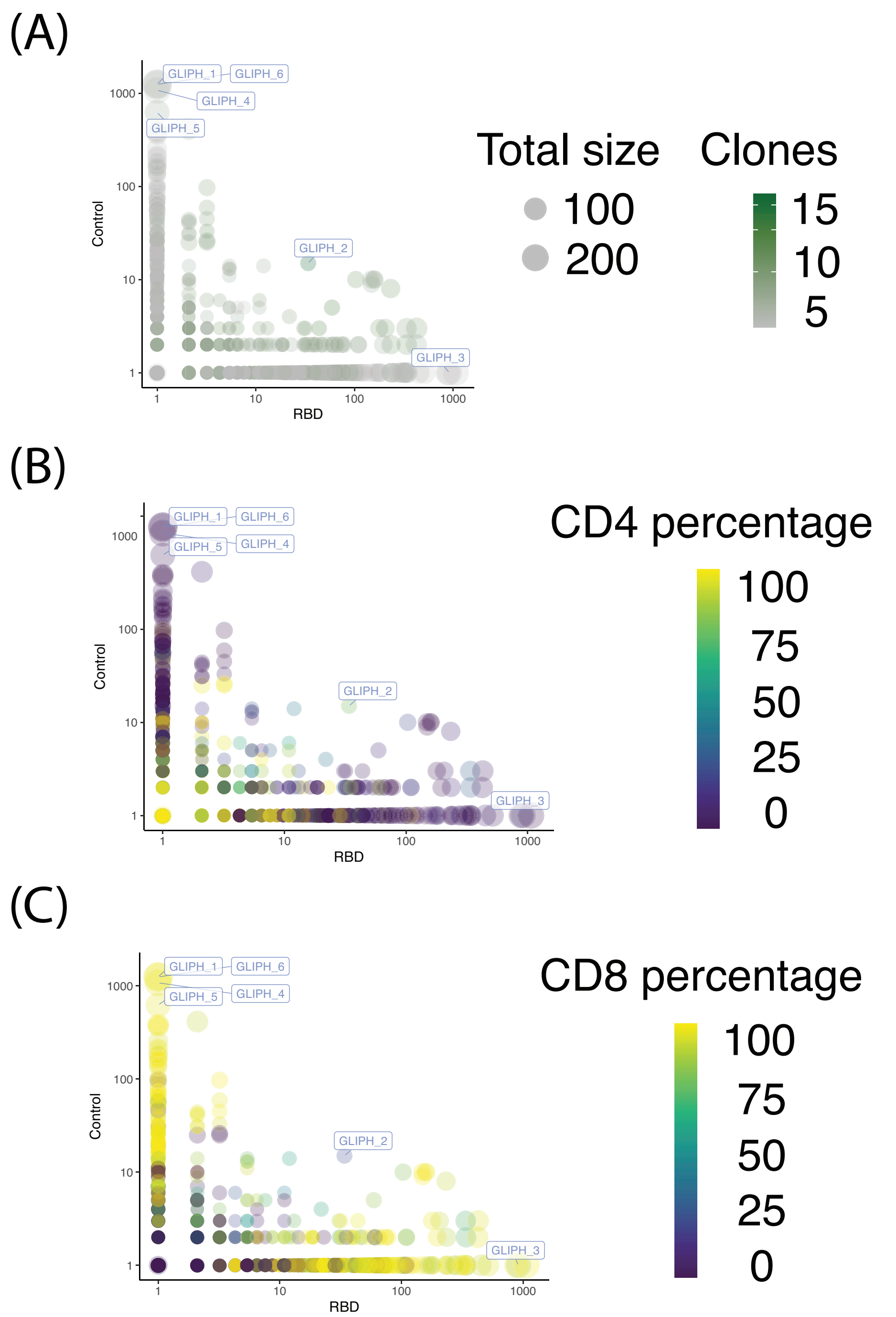

Supplementary Figure 6: **GLIPH2 cluster details on the MS data.** Scatter plots comparing GLIPH2 clusters between healthy control and patients with MS. GLIPH2 clusters are sized according to the number of cells in which the given specificity group is observed, and colored according to the number of unique clonotypes that belong to a given cluster (A), percentage of cells within given cluster assigned to the CD4+ T cell group (B), and percentage of cells within given cluster assigned to the CD8 T Cell group (C). The selected clusters are labeled with their names in blue.

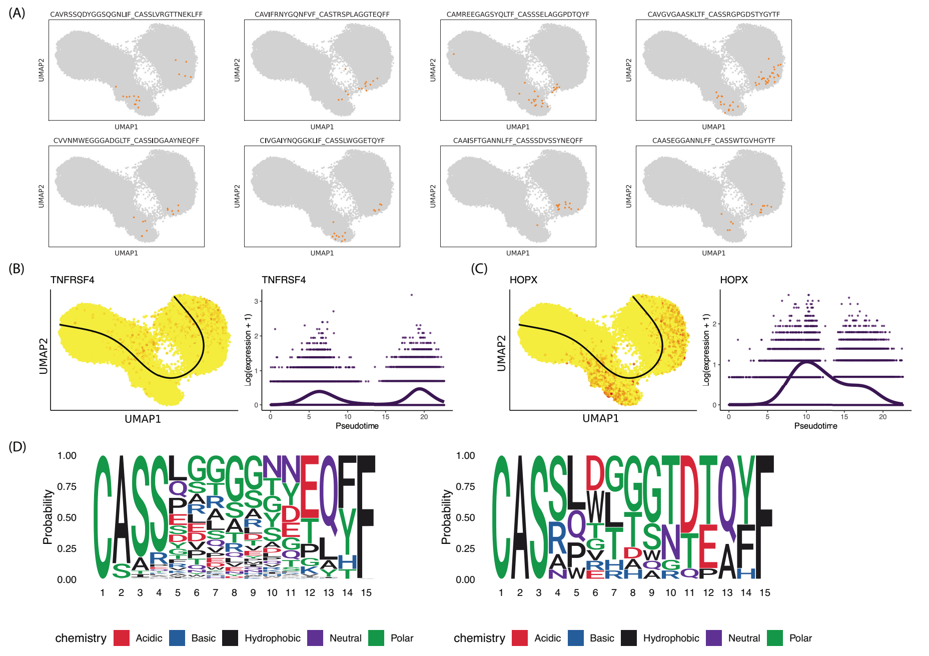

Supplementary Figure 7: **TCR and RNA features in the MS blood and CSF data.** (A) Clonotypes shared between blood and CSF. (B-C) More genes are associated with the trajectory identified by TradeSeq in UMAP (left) and the corresponding smoothers (right). (D) Distribution of the CDR3 β chain in the transitioning cell cluster (right) and random T cells of the same size (left).

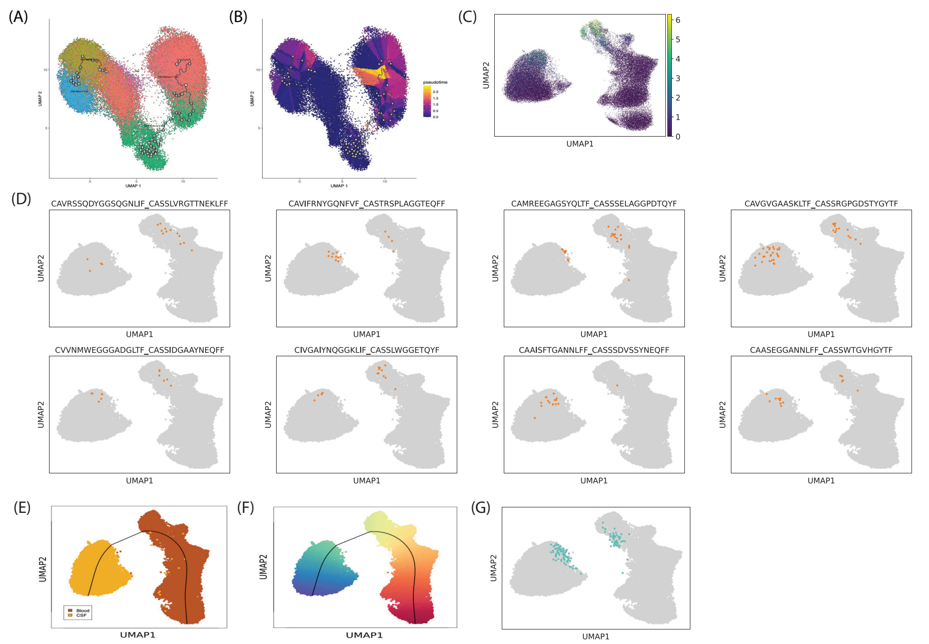

Supplementary Figure 8: **Monocle3 results on scNAT data and mapping TCR features onto RNA space.** (A) Principal graph inferred by Monocle3. (B) Monocle3 pseudotime based on the principal graph. (C) RNA UMAP colored by expansion score. (D) Clonotypes shared between blood and CSF on RNA UMAP. (E-F) Slingshot inferred trajectory colored by tissue (E) and pseudotime (F) using RNA data only. (G) The cluster of T cells that are in transitioning state mapped onto the RNA UMAP space.
