# Supplementary material for: scNAT: a deep learning method for integrating paired single-cell RNA and T cell receptor sequencing profiles
Source: Genome Biol. 2023 Dec 18;24:292. doi: 10.1186/s13059-023-03129-y (PMC10726524; doi:10.1186/s13059-023-03129-y)
Supplement: Supplementary file 5 — Additional file 5. Review history. [file 13059_2023_3129_MOESM5_ESM.docx]

**1^st^ round**

**Reviewer 1**

The manuscript proposes a deep-learning-centered computational method that will integrate information from paired scRNA-seq and scTCR-seq data. The method's efficiency is primarily assessed based on its ability to identify new cell types and infer trajectories compared to the use of a single modality alone. The manuscript's strength lies in its application to "paired scRNA-seq and scTCR-seq data"; however, the innovation in methodology is less compelling. Specific comments are as follows:

1. The manuscript is written clearly and provides a detailed description of scTCR-seq data. However, it lacks a clear explanation of the unique data science challenges inherent in integrating scRNA-seq and scTCR-seq data, that is, challenges not already addressed in CITE-seq or scRNA-scATAC-seq integration.

2. Following from point 1. In the discussion the author mentioned "While we only explore the integration of the TCR and RNA data, this framework can be extended to include other data modalities such as single-cell ATAC-seq[38] and CITE-seq[39] cell surface protein information." It important to note that there are a series of methods that can handle integration of both paired and unpaired data. It is important to clearly define the technical innovation of scNET.

3. The introduction will benefit from a clear articulation of the contribution of this manuscript. The author should clearly layout either one of the options below
The integration with scTCR-seq has addressed a new computational challenge that is unique to these two data type.
There is new computational extension that is new compared to existing deep-learning based integration approaches.

4. While the paper demonstrate the additional information provided by jointly examine scRNA-seq and scTCR-seq data. The current manuscript didn't demonstrate whether existing approaches such as MOFA2, GLUE, scJoint, scAI, multiVI, Seurat and many other similar methods can be applied here, and will they provide similar biological results when applied to the MS data. Note, that while some methods were designed on "unpaired" data, it doesn't limit user to apply those methods to paired data.
5. The claim on "scNAT can further remove remaining batch effects" is rather strong given the demonstration is only against ResPAN. May be a simulation study is able to articulate this component better. How is scNAT performance if no normalization is done a-prior ? Alternatively, a small comparison with popular data integration approaches may be more appropriate before making such statement.

Code and Software Review:
5. The repository appears to be well organized, with each section related to a specific aspect of the "scNAT" project. The code appears to be reasonably clean, and the detailed comments could improve the code's readability and make it easier for users to understand its functionality. From the code, the overall neural network architecture is consistent with what the authors claimed in their illustration.

However, the repository does not contain a dedicated test suite. The tutorial provided in it contains invalid data path of TCR file (as "/dir/to/tcr/file/") and single-cell RNAseq file (as "/dir/to/rna/file/rna.csv"). Meanwhile, the repository does not provide any test data or data link, which causes much inconvenience for result repetition and comparison. In shoft the tutorial (https://github.com/biqing-zhu/scNAT/blob/main/tutorials/scNAT_tutorial.ipynb) is simplistic with lots of warning messages and the paper will benefit from a genius effort towards community sharing of results.

**Reviewer 2**

This manuscript presented a method to integrate paired scRNA-seq and scTCR-seq data to derive a unified latent space for downstream analysis. scNAT is claimed to be able to remove batch effects, while keeping both RNA and TCR modality information. Application of scNAT to multiple sclerosis data identified a intermediate T cell phenotype during the migration from blood to cerebrospinal fluid. Overall, integration of different types of genomics data for improved single cell clustering is a desirable task. Concerns with this method include its incremental contributions over the existing approaches, insufficient data analyses, and lack of key details of the method.

Specifically,
1) The methodology novelty is unclear. The authors used a typical VAE architecture to encode both gene expression and TCR sequencing data. The network structure related to TCR embedding is entirely inherited from another work, DeepTCR while removing D gene usage. In another recent work, Zhang et al. [PMID:33408405] proposed a method called Tessa to integrate TCRs with gene expression of T cells to estimate the effect that TCRs confer on the phenotypes of T cells. They also used a VAE architecture to encode TCRs. The authors need to provide a detailed comparison with Tessa for the downstream data analysis tasks. For example, can Tessa also uncover the transitional T cell phenotype from the MS dataset? How about batch effects and classification accuracy? The authors need to clearly discuss their advantages over DeepTCR, Tessa and CoNGA.

2) The authors applied scNAT to identify a cell cluster which is in the transitioning state between blood and CSF, and suggested that these cells were migrating from blood to CSF and were clonally expanded due to activation. A standard alternative approach is to repeat the trajectory analysis using only scRNA-seq data, and overlay TCR sequences to these cells to get their clonal expansion information. Can this simple approach also (partially) uncover the cell cluster reported by the authors? To date, there is no clear biological evidence to support that this cluster is valid.

3) About the motif results of the small transitioning cluster, the authors showed more homogeneous motif of this cluster than random cells. How many random cells did the authors choose? One can get a heterogeneous motif when using a large number of cells.

4) The method section lacked detailed information of some algorithms/methods. For instance, how to get the UMAP plot based on scTCR-seq data only? How was the expansion score calculated? How was the sicelltype calculated in the ASW formula?

5) About the CNN layers of the CDR3 sequence encoder, the authors stated that they used 32, 64 and 128 filters, however, the dimension of final CDR3 features is 64 for one chain in supplementary figure 1C.

6) About the selection of loss weight parameters, it is not clear why the authors selected the combination. It didn't achieve the best Accuracy + ASW score.

7) There are typos throughout the manuscript. Some figures are mislabeled. For instance, on page 4 line 34, it should be 'Supplementary Figure 5', so are lines 45 and 57. On page 8 line 18, what is 'NSC'? Should it be ASW?

**Authors’ response**

Reviewer: 1

The manuscript proposes a deep-learning-centered computational method that will integrate  information from paired scRNA-seq and scTCR-seq data. The method's efficiency is primarily  assessed based on its ability to identify new cell types and infer trajectories compared to the  use of a single modality alone. The manuscript's strength lies in its application to "paired  scRNA-seq and scTCR-seq data"; however, the innovation in methodology is less compelling.  Specific comments are as follows:

1. The manuscript is written clearly and provides a detailed description of scTCR-seq data.  However, it lacks a clear explanation of the unique data science challenges inherent in  integrating scRNA-seq and scTCR-seq data, that is, challenges not already addressed in CITE seq or scRNA-scATAC-seq integration.

We thank you for bringing up this issue and we apologize for not clearly stating the challenges  in integration scRNA-seq and scTCR-seq in the previous version of the manuscript. The main  differences between scTCR-seq data and other multi-omics data types are that scTCR-seq data are not numerical. More specifically, the CDR3 part is considered as text data and the V/(D)/J  gene part is categorical data in machine learning. Thus, methods that have been developed for  integrating CITE-seq or scRNA-scATAC-seq data can not be applied to this problem because  they are designed specifically for numerical data. The main novelty of our method lies in its  ability to handle non-numerical data. We have added more details to the third paragraph in the  Background section on page 2, the first paragraph in the Discussion section on page 5, and the  Conclusion section on page 6.

2. Following from point 1. In the discussion the author mentioned "While we only explore the  integration of the TCR and RNA data, this framework can be extended to include other data  modalities such as single-cell ATAC-seq[38] and CITE-seq[39] cell surface protein information."  It important to note that there are a series of methods that can handle integration of both paired  and unpaired data. It is important to clearly define the technical innovation of scNET.

Thank you for raising this point. We agree that there are well-known methods designed for the integration of scRNA-seq with ATAC-seq and CITE-seq, which considered these data as  numerical. A better extension and application of scNAT should lie in multi-omics containing non numerical data, such as scRNA-seq and scBCR-seq data, as well as other 10x Single Cell  Immune Profiling, for example, TCR, RNA, and cell surface marker. We have updated the  corresponding statement in the second paragraph of the Discussion section on page 6.

3. The introduction will benefit from a clear articulation of the contribution of this manuscript. The  author should clearly layout either one of the options below

The integration with scTCR-seq has addressed a new computational challenge that is unique to  these two data type.

There is new computational extension that is new compared to existing deep-learning based  integration approaches.

Thank you for your suggestion. The new computational challenge that is unique to these two  data types is similar to our response to your first point. Specifically, the multi-omics data  integration methods so far only focus on numerical data, whereas scNAT can handle additional  complex data types, including CDR3 and V/(D)/J gene which are text and categorical data. To  the best of our knowledge, scNAT is the first method that can integrate numerical and non numerical data and embed them into a united latent space that facilitates downstream analysis.

4. While the paper demonstrate the additional information provided by jointly examine scRNA seq and scTCR-seq data. The current manuscript didn't demonstrate whether existing  approaches such as MOFA2, GLUE, scJoint, scAI, multiVI, Seurat and many other similar  methods can be applied here, and will they provide similar biological results when applied to the  MS data. Note, that while some methods were designed on "unpaired" data, it doesn't limit user  to apply those methods to paired data.

Thank you for the suggestions. As mentioned above, those methods only work for numerical  data, and are not able to handle TCR data containing CDR3 and V/(D)/J gene features, which  are considered as text and categorical data types, respectively.

5. The claim on "scNAT can further remove remaining batch effects" is rather strong given the  demonstration is only against ResPAN. May be a simulation study is able to articulate this  component better. How is scNAT performance if no normalization is done a-prior? Alternatively,  a small comparison with popular data integration approaches may be more appropriate before  making such statement.

Thank you for the suggestion of considering other methods. Our main purpose for this section is  to show that we recommend to use batch-corrected data (such as ResPAN output) as input to  scNAT because we did not specifically design any batch correction module for scNAT, but from  the scNAT output, we still observed a slight batch correction improvement on top of the ResPAN  result.

Following your suggestion, we experimented on some other RNA batch correction methods  using the MS RNA data, including Seurat v4, MNN, as well as BBKNN, and we summarized the  results in Table 1 in the manuscript. scNAT gave the highest 1 – bASW and 1 – kBET scores  among all the methods, further demonstrating its good performance.

Code and Software Review:

5. The repository appears to be well organized, with each section related to a specific aspect of  the "scNAT" project. The code appears to be reasonably clean, and the detailed comments  could improve the code's readability and make it easier for users to understand its functionality.  From the code, the overall neural network architecture is consistent with what the authors  claimed in their illustration.

However, the repository does not contain a dedicated test suite. The tutorial provided in it  contains invalid data path of TCR file (as "/dir/to/tcr/file/") and single-cell RNAseq file (as  "/dir/to/rna/file/rna.csv"). Meanwhile, the repository does not provide any test data or data link,  which causes much inconvenience for result repetition and comparison. In shoft the tutorial  (https://nam12.safelinks.protection.outlook.com/?url=https%3A%2F%2Fgithub.com%2Fbiqing zhu%2FscNAT%2Fblob%2Fmain%2Ftutorials%2FscNAT_tutorial.ipynb&data=05%7C01%7Ch ongyu.zhao%40yale.edu%7C75eea198107c4fd79c9508dbab0546d7%7Cdd8cbebb21394df8b4 114e3e87abeb5c%7C0%7C0%7C638291811383688986%7CUnknown%7CTWFpbGZsb3d8ey JWIjoiMC4wLjAwMDAiLCJQIjoiV2luMzIiLCJBTiI6Ik1haWwiLCJXVCI6Mn0%3D%7C3000%7C%

7C%7C&sdata=ChHyp9PjGfGwzCjrWoE62pDkSD%2FnSjH8Bd64IdFSQa8%3D&reserved=0)  is simplistic with lots of warning messages and the paper will benefit from a genius effort  towards community sharing of results.

Thank you for your careful review of our repository and great suggestions. We have deposited a  sample dataset with 4,000 cells (https://github.com/biqing-zhu/scNAT/tree/main/data), and  updated the data paths of the TCR file and scRNA-seq file as you suggested. We have also  cleaned up the warning messages in the tutorial (https://github.com/biqing zhu/scNAT/tree/main/tutorials). We hope the tutorial is now cleaner and easier for users to  follow.

Reviewer 2:

This manuscript presented a method to integrate paired scRNA-seq and scTCR-seq data to  derive a unified latent space for downstream analysis. scNAT is claimed to be able to remove  batch effects, while keeping both RNA and TCR modality information. Application of scNAT to  multiple sclerosis data identified a intermediate T cell phenotype during the migration from blood  to cerebrospinal fluid. Overall, integration of different types of genomics data for improved single  cell clustering is a desirable task. Concerns with this method include its incremental  contributions over the existing approaches, insufficient data analyses, and lack of key details of  the method.

Specifically,

1) The methodology novelty is unclear. The authors used a typical VAE architecture to  encode both gene expression and TCR sequencing data. The network structure related to TCR  embedding is entirely inherited from another work, DeepTCR while removing D gene usage. In  another recent work, Zhang et al. [PMID:33408405] proposed a method called Tessa to  integrate TCRs with gene expression of T cells to estimate the effect that TCRs confer on the  phenotypes of T cells. They also used a VAE architecture to encode TCRs. The authors need to  provide a detailed comparison with Tessa for the downstream data analysis tasks. For example,  can Tessa also uncover the transitional T cell phenotype from the MS dataset? How about  batch effects and classification accuracy? The authors need to clearly discuss their advantages  over DeepTCR, Tessa and CoNGA.

We thank you for your comments, questions, and suggestions. We have included the limitations  of DeepTCR, tessa, and CoNGA to the third paragraph of the Background section on page 2 in  our updated manuscript. Specifically, DeepTCR does not include RNA data which contain transcription-level information. CoNGA incorporates RNA data but it still visualizes RNA and  TCR data through separate UMAP representations. Moreover, it aggregates all the cells within  the same clonotype, resulting in a loss of single-cell-level resolution. Tessa, on the other hand,  does not take into consideration the CDR3α and V/(D)/J gene information, and like CoNGA,  makes an unrealistic assumption that all cells with the same clonotype share identical  transcriptomic profiles.

Following your suggestion, we implemented tessa with default parameters and studied its  output, including (1) TCR networks and (2) weighted TCR embedding. We were not able to  finish running tessa on our full dataset with 48,898 cells in three days, so due to tessa’s high  computational cost, we subsampled 25% of the cells, which cost 8.47G memory and 21 hours of  computation time. The TCR network is shown in Figure A below. There is no obvious pattern in  the figure to reflect biological information. Figures B and C show the UMAP calculated by the  weighted TCR embedding colored by either cell type or tissue. Again, tessa was not able to  retain any cell type-specific or tissue-specific information. We also ran DeepTCR on our dataset.  The results (Figures D and E) are similar to scNAT with TCR only data, with no cell type or

tissue information preserved. This is expected since the structure of the TCR part of scNAT is  similar to DeepTCR.

In conclusion, we have further demonstrated that by integrating scRNA-seq and scTCR-seq  data into a united latent space, scNAT can achieve better performance in terms of cell type and  tissue information preservation.

2) The authors applied scNAT to identify a cell cluster which is in the transitioning state  between blood and CSF, and suggested that these cells were migrating from blood to CSF and  were clonally expanded due to activation. A standard alternative approach is to repeat the  trajectory analysis using only scRNA-seq data, and overlay TCR sequences to these cells to get  their clonal expansion information. Can this simple approach also (partially) uncover the cell  cluster reported by the authors? To date, there is no clear biological evidence to support that  this cluster is valid.

Thank you for your suggestion, and we think it is a great idea to repeat the trajectory analysis  using scRNA-seq data only, and have added the results to Supplementary Figure 6. We found  that the RNA data can partially reflect the cell migration pattern, by showing the cells with high  expansion scores on the top of blood and CSF (Figure A), as well as the pseudotime and  trajectory (Figures C/D), similar to Figure 4(A). In addition, the largest clones that are shared  between the two tissues reside in similar positions as with scNAT integrated data (Figure B, and  Supplementary Figure 5(A)). However, the cells that were identified as being in transitioning  state are not in the middle of the two tissues. Instead, they were pushed back to the boundaries  of blood and CSF (Figure E). Nevertheless, they are still close to each other, indicating their  similarity. In conclusion, the overall trajectory pattern identified by scNAT can be supported by  RNA data, but RNA data can only partially reflect this migration since they can neither bridge  the two tissues nor identify the cells that are in transition state.

3) About the motif results of the small transitioning cluster, the authors showed more  homogeneous motif of this cluster than random cells. How many random cells did the authors  choose? One can get a heterogeneous motif when using a large number of cells.

We apologize that we did not make this clear in the original manuscript. The number of the  random cells we used to compute the motif result is the same as that for the transitioning cluster  (n = 716). We have also added this detail to the *Characterization of the T cell trafficking  trajectory from blood to CSF* section on page 5 of the revised manuscript.

4) The method section lacked detailed information of some algorithms/methods. For  instance, how to get the UMAP plot based on scTCR-seq data only? How was the expansion  score calculated? How was the sicelltype calculated in the ASW formula?

We apologize for the lack of these details in our original submission. For the scTCR-seq UMAP,  we only input scTCR-seq data to scNAT (*include_RNA = False* in the model), and then we used  the latent space data for UMAP calculation similar to RNA + TCR data. The expansion score is  defined the same way as that in Pappalardo et al., which is the log2 of the number of cells in a  clonal group and corresponds to clone size. For the cell-level ASW score, it is defined as:  ^(��)^ =^��(��)−��(��)^ 

��_����������������_

max {��(��)_,��(��)}_, where *i* represents each cell, *a(i)* is the mean distance from cell *i* to all  

other cells that belong to the same cell type, *b(i)* is the lowest average distance from cell *i* to all  cells in the same cell type among all other cell types. We have also added the descriptions for  ASW and TCR UMAP under the Methods section on page 8 and 9 under *Grid search for  parameter tuning* and *UMAP calculation*, and expansion score under the section  *Characterization of the T cell trafficking trajectory from blood to CSF* on page 4.

5) About the CNN layers of the CDR3 sequence encoder, the authors stated that they used  32, 64 and 128 filters, however, the dimension of final CDR3 features is 64 for one chain in  supplementary figure 1C.

Thank you for pointing this out. Indeed, the dimension of the concatenated CDR3 vector was  mislabeled in Supplementary Figure 1(C). We have updated the figure, with the corrected  dimension being ‘Concatenate (768 * 2)’. Specifically, the dimension after the first filter is (1, 50,  32), after the second filter is (1, 17, 64), and after the third filter is (1, 6, 128). Finally, we  flattened the tensor, resulting in a vector of dimension 768 for each chain. We hope this is  clearer now.

6) About the selection of loss weight parameters, it is not clear why the authors selected the  combination. It didn't achieve the best Accuracy + ASW score.

We apologize that we missed another criterion under *Grid search for parameter tuning* in the  methods section of the original submission on page 9. Apart from ASW > 0.5, we only selected the parameters whose corresponding loss did not exceed 115% of the minimal loss so that the  model fitness will not be sacrificed. The following bar plots show the loss increase ratio under  different *alpha_latent* and *alpha_gene* across *alpha_seq*. Specifically, each bar was calculated  by dividing the mean loss under the corresponding *alpha_latent* and *alpha_gene* across all  *alpha_seq*, by the minimum loss across all *alpha_seq* to reflect the loss ratio under different  settings. These results show that regardless of *alpha_latent* and *alpha_seq*, *alpha_gene* = 1e-2  always has much higher loss increase compared with other *alpha_gene*, and that is the reason  why we did not select *alpha_gene* = 1e-2.

7) There are typos throughout the manuscript. Some figures are mislabeled. For instance, on  page 4 line 34, it should be 'Supplementary Figure 5', so are lines 45 and 57. On page 8 line 18,  what is 'NSC'? Should it be ASW?

We sincerely apologize for the typos. We have updated the names from 'Supplementary Figure  4’ to 'Supplementary Figure 5’ on the three lines you mentioned, and also switched ‘NSC’ to  ‘ASW’ so that the naming is more consistent.

**2nd round**

**Reviewer 1**

I thank the authors for their responses, and the newly revised manuscript is much improved.

While the authors have answered all of the questions raised in the previous review, I have one additional point to clarify. In authors’ response to #5 regarding scNAT and its impact of the batch effect is appreciated. However, the concept that they recommend the use of scNAT post-normation didn’t really come out in their current Results section. I like to see the authors incorporate the essence of their response (e.g recommendation of using scNAT post-normalization) clearly into their manuscript in the revised version.

**Reviewer 2**

The new analysis are important and necessary to lift the potential confusions regarding novelty. The authors need to include comparisons and discussions of other methods, including Tessa, DeepTCR and CoNGA in the manuscript. In addition, it seems that scNAT is computationally efficient -- please include a comparison of run time + memory in the paper. Comparisons with monocle pseudotime trajectory should also be included. I don't have further comments.

**Authors’ response**

Reviewer: 1

While the authors have answered all of the questions raised in the previous review, I

have one additional point to clarify. In authors' response to #5 regarding scNAT and its

impact of the batch effect is appreciated. However, the concept that they recommend

the use of scNAT post-normation didn't really come out in their current Results section.

I like to see the authors incorporate the essence of their response (e.g

recommendation of using scNAT post-normalization) clearly into their manuscript in the

revised version.

We thank you for your comments and suggestions. We have added scNAT with RNA

raw counts as input to the section scNAT latent features represent information from

both the scRNA-seq as well as the scTCR-seq data on page 4, and the Training VAE

Methods section on page 9. The plots can be found in supplementary figure 4B.

Specifically, the RNA raw counts have extremely large values (the maximum value

being 778) which resulted in infinite weights during training, and therefore we had to

stop the training after two epochs. The resulting UMAP plots lack the ability to reflect

cell type or tissue information, likely due to the underfitting caused by the early

stopping. The results show the importance of data normalization before inputting to

scNAT.

Reviewer #2: The new analysis are important and necessary to lift the potential

confusions regarding novelty. The authors need to include comparisons and

discussions of other methods, including Tessa, DeepTCR and CoNGA in the

manuscript. In addition, it seems that scNAT is computationally efficient -- please

include a comparison of run time + memory in the paper. Comparisons with monocle

pseudotime trajectory should also be included. I don't have further comments.

We thank you for bringing up this issue and we apologize for not adding the

comparison to the manuscript. We have incorporated the comparison with tessa,

DeepTCR, as well as CoNGA into the section scNAT latent features represent

information from both the scRNA-seq as well as the scTCR-seq data on page 4, as

well as the Discussion on page 6. The run time and memory comparison were added

to supplementary table 2. Following your suggestion, we also performed Monocle3

trajectory analysis, and the results were added to the section Characterization of the T

cell trafficking trajectory from blood to CSF on page 5 and supplementary figure 8(A-B).

Monocle3 identified a similar pattern as Slingshot, with a trajectory from blood to CSF.

This helps further validate our finding that T cells can transmigrate from blood to CSF

for immune surveillance.
